# Oropharyngeal dysphagia and gastroesophageal reflux disease in lung transplant patients: a systematic review and meta-analysis of incidence, risk factors, and clinical outcomes

**DOI:** 10.7717/peerj.21472

**Published:** 2026-07-06

**Authors:** Yuanli Tang, Feiyao Deng, Jing Pan

**Affiliations:** Department of Respiratory and Critical Care Medicine, West China Hospital, Sichuan University, Chengdu, Sichuan Province, China

**Keywords:** Oropharyngeal dysphagia, Gastroesophageal reflux disease, Cystic fibrosis, Bronchiolitis obliterans syndrome, Lung transplantation

## Abstract

**Background:**

This systematic review and meta-analysis aimed to quantitatively synthesize the existing evidence to determine the prevalence of oropharyngeal dysphagia (OPD) and gastroesophageal reflux disease (GERD) in lung transplant recipients, identify key risk factors for OPD, and evaluate the impact of GERD on critical clinical outcomes.

**Methods:**

Strategic searches of several electronic databases such as PubMed, Embase, the Cochrane Library, and Web of Science were performed to find the relevant studies in accordance with the Preferred Reporting Items for Systematic Reviews and Meta-Analyses (PRISMA) guidelines from their inception until December 2025. A random-effects model was applied to compute the prevalence rates. Synthesis of risk factors of OPD and correlation of GERD and clinical outcomes were assessed. The Newcastle–Ottawa Scale was used to evaluate the risk of bias.

**Results:**

A total of 35 studies with 5,245 patients who underwent lung transplantation were included. The analysis revealed that OPD and GERD are highly prevalent after lung transplantation, with pooled prevalence rates of 48% (95% CI [0.41–0.55]) and 43% (95% CI [0.35–0.52]), respectively. Significant risk factors for OPD included mechanical ventilation (OR = 5.37, 95% CI [3.25–8.86]) and reintubation (OR = 2.76, 95% CI [1.29–5.90]). Moreover, the incidence of cystic fibrosis (CF) was significantly higher in patients with GERD compared to non-GERD patients (RR = 1.70, 95% CI [1.16–2.49]). Most importantly, GERD was established as a significant risk factor for the development of bronchiolitis obliterans syndrome (BOS, HR = 2.89, 95% CI [1.35–6.17]).

**Conclusion:**

OPD and GERD are prevalent and influence comorbidities among the lung transplant patients. The discovery of particular, modifiable risk factors of OPD and the validation of the importance of GERD in chronic allograft dysfunction contribute to the evidence of the paramount role of preventive screening and management approaches towards both diseases in enhancing patient outcomes.

## Introduction

Lung transplantation a definitive surgical intervention for patients with end-stage pulmonary conditions (Patti et al., 2016), including chronic obstructive pulmonary disease (COPD) (Perch et al., 2022), idiopathic pulmonary fibrosis (IPF) (Hernandez et al. 2018), and CF (Yeung et al., 2020). More than 4,600 lung transplants are performed annually worldwide, with bilateral procedures accounting for approximately 80% of cases, predominantly in North America (55%) and Europe (36%) (Chambers et al., 2018). Since the first lung transplant in 1963, advances in surgical technique, immunosuppressive therapy, and postoperative care have substantially improved outcomes, leading to enhanced survival and quality of life for recipients (Van der Mark, Hoek & Hellemons, 2020). Over the last decades, median survival following lung transplantation has improved significantly, increasing from 4.3 years (1990–1998) to 6.5 years (2009–2016). Current median survival is 6.2 years globally, with 8.3 years among patients surviving the first year (Khush et al., 2018). Despite these improvements, lung transplantation continues to have a higher one-year mortality rate than other solid organ transplants (Foroutan et al., 2022). Major contributors to mortality include infectious complications (DeFreitas et al., 2021), allograft rejection and dysfunction (Beeckmans et al., 2023), and bronchiolitis obliterans syndrome (BOS) (Weigt et al., 2013). Among infectious etiologies, bacterial pneumonia remains a leading cause, often resulting from aspiration of oral secretions in the setting of oropharyngeal dysphagia (OPD) or micro-aspiration of gastric contents due to gastroesophageal reflux disease (GERD) (Black et al., 2021; Miles et al., 2021).

Major contributors to post-transplant mortality include infectious complications, allograft rejection and dysfunction, and bronchiolitis obliterans syndrome. Among infectious etiologies, bacterial pneumonia remains a leading cause, often resulting from aspiration of oral secretions in the setting of OPD or micro-aspiration of gastric contents due to GERD (Liu et al., 2024; Righi et al., 2024). Both conditions (OPD and GERD) are well-recognized complications following thoracic surgery, particularly lung transplantation (Atkins et al., 2007; Hathorn, Chan & Lo, 2017). OPD defined as impairment in the oral, pharyngeal, or upper esophageal phases of swallowing, is a frequent postoperative complication that predisposes patients to aspiration pneumonia, prolonged hospitalization, and increased mortality (Atkins et al., 2007; Baumann et al., 2017; Herrera et al., 2006). Early identification and management of dysphagia have been shown to improve outcomes in other high-risk populations, such as stroke survivors (*e.g.*, stroke (Palli et al., 2017; Park, Kim & Lee, 2020)) and critically ill patients (Troll et al., 2023), suggesting that similar protocols may be beneficial in lung transplant recipients. Concurrently, GERD is highly prevalent both before and after lung transplantation. Among patients with end-stage lung disease, reflux symptoms are common, and objective testing reveals significant acid reflux in a substantial proportion of cases, often in the absence of symptoms (Savarino et al., 2013). This is particularly pronounced in patients with IPF, who exhibit higher rates of total and proximal reflux episodes compared to other lung disease cohorts or healthy controls. Following transplantation, the prevalence of gastroesophageal reflux disease increases markedly, with reported rates ranging from 51% to 69% (Davis et al., 2010; Hadjiliadis et al., 2003). Importantly, GERD is not merely a comorbid condition but a direct contributor to adverse outcomes. It has been established as an independent risk factor for BOS, the most common phenotype of chronic lung allograft dysfunction (King et al., 2009). BOS develops in up to 50% of lung transplant recipients within the first five years and represents the leading cause of late mortality (Kulkarni et al., 2019; Nykänen et al., 2020). These findings underscore the critical need for systematic screening and management of both OPD and GERD in the lung transplant population.

Despite the well-recognized risks associated with these conditions (Banda et al., 2022; Eusebi et al., 2018; Song et al., 2024), systematic characterization of their epidemiology, risk factors, and clinical impact in lung transplant recipients remains limited. Existing studies report widely varying incidence estimates, reflecting heterogeneity in diagnostic criteria, study populations, and follow-up durations. Large-scale, population-based estimates using standardized definitions are lacking, and the true incidence in contemporary transplant cohorts is not well established. Accordingly, this meta-analysis aims to: (1) evaluate the pooled prevalence of OPD and GERD in lung transplant recipients; (2) identify risk factors associated with these conditions based on the existing literature; and (3) assess the clinical outcomes associated with these complications in this patient population.

## Methods & Methods

This systematic review and meta-analysis were conducted in line with the Preferred Reporting Items for Systematic Reviews and Meta-Analyses (PRISMA) guidelines (Page et al., 2021), and the completed checklist is presented in Table S1. The study protocol was registered with the International Prospective Register of Systematic Reviews (PROSPERO; registration number: CRD420251069190).

### Inclusion and exclusion criteria

The inclusion criteria were as follows: (1) cohort trials on audlt patients (aged ≥18 years) who had undergone lung transplantation; (2) trials that have reported the prevalence of OPD and GERD. OPD was defined as any impairment in the oral or pharyngeal phases of swallowing affecting the safe or efficient transit of a bolus from the mouth to the esophagus, diagnosed by clinical swallowing assessment, videofluoroscopic swallowing study, fiberoptic endoscopic evaluation of swallowing, or other validated instrumental or clinical assessments as reported. GERD was defined based on typical symptoms confirmed by endoscopic examination or 24 h esophageal pH measurement; (3) trials that identified potential risk factors leading to OPD (*e.g.*, age, body surface area (BSA), GERD, Mechanical ventilation, and reintubation), or trials that have evaluated the frequency of clinical events (*e.g.*, COPD, IPF, or CF), or trials that have evaluated the association between GERD and post-transplant outcomes (*e.g.*, CLAD, BOS and mortality); the studies were excluded when they were review articles, case reports, animal experiments or when the necessary data could not be obtained in the published article.

Exclusion criteria were as follows: (1) studies that included pediatric patients (aged < 18 years) or that did not separately report data for adult patients were excluded; (2) non-cohort study designs, including review articles, case reports, case series, editorials, commentaries, conference abstracts, and animal or experimental studies, were also excluded. (3) Studies that did not assess OPD or GERD as an exposure, or did not report outcomes related to OPD/GERD were excluded; (4) duplicate or overlapping publications from the same cohort were excluded, retaining the study with the largest sample size or most complete data where applicable.

### Search strategy

We performed a systematic literature search of PubMed, Embase, the Cochrane Library, and Web of Science (WoS) from their inception until December 2025, with no language restrictions applied. The search aimed to identify studies reporting the prevalence and associated factors of OPD and gastroesophageal reflux disease GERD in patients following lung transplantation. The strategy combined Medical Subject Headings (MeSH) terms, including ‘Lung Transplantation’, ‘Deglutition Disorders’, and ‘Gastroesophageal Reflux’, with corresponding free-text keywords. Boolean operators (OR, AND) were uniformly applied across all databases to merge the search terms. These terms were systematically combined using Boolean operators (OR within concepts, AND between concepts) to optimize retrieval. The full search strategy for PubMed is available in Table S2, and analogous strategies were employed for other databases. To ensure comprehensive coverage, the reference lists of all included studies were also manually screened for additional relevant publications.

### Study selection and data extraction

Following the criteria for inclusion and exclusion outlined previously, two investigators (Feiyao Deng and Jing Pan) independently selected studies based on the predefined eligibility criteria. After removing duplicates in EndNote X9, the titles and abstracts of all identified records were screened for relevance. The full texts of potentially eligible studies were then retrieved and assessed. Any disagreements were resolved through discussion or by consulting a third researcher (Yuanli Tang).

Two authors (Feiyao Deng and Jing Pan) independently extracted data using a standardized form adapted from the Cochrane template. The extracted information included: (1) basic study characteristics (title, first author, publication year, region, design); (2) participant characteristics (sample size, age, sex, diagnostic criteria for OPD and GERD); and (3) outcome data (incidence of OPD and GERD, relevant risk factors). Any discrepancies were resolved through consensus or by consulting a third author (Yuanli Tang).

### Quality assessment of the included studies

To evaluate potential bias and the overall quality of the included studies, we employed the Newcastle–Ottawa Scale (NOS), a widely utilized tool for assessing the methodological quality or risk of bias in cohort studies, according to Cochrane recommendations (Lo, Mertz & Loeb, 2014; Luchini et al., 2021; Stang, 2010). The evaluation of publication bias in the included studies was independently conducted by two researchers (Feiyao Deng and Jing Pan), with any discrepancies resolved through consultation or discussion with a third researcher (Yuanli Tang). Each included study underwent an evaluation based on specific criteria. The criteria consisted of selection (scored from 0 to 4), comparability (scored from 0 to 2), and outcome (scored from 0 to 3). The results were then interpreted according to commonly accepted standards and categorized into the following groups: very high risk of bias (0–3 NOS points), high risk of bias (4–6 NOS points), and low risk of bias (7–9 NOS points) (Lo, Mertz & Loeb, 2014).

### Data analysis

All statistical analyses were performed using STATA version 16.0. Given the expected heterogeneity across studies, pooled prevalence estimates for OPD and GERD were calculated using a random-effects (DerSimonian and Laird) model). For the comparison of clinical event incidence between GERD and non-GERD patients, risk ratios (RRs) with 95% confidence intervals (CIs) were computed. The association between potential risk factors and OPD was expressed as odds ratios (ORs) with 95% CIs, and the predictive value of GERD on clinical outcomes was expressed as harzd ratios (HRs) with 95% CIs; these ORs/HRs were log-transformed and pooled using the generic inverse-variance method under a random-effects model for consistency. We performed the *I*^2^ test to determine the heterogeneity across the included studies. An *I*^2^ value exceeding 50% indicated a significant degree of heterogeneity among the included studies. Regarding the inevitable heterogeneity, we used random-effect models for all the analyses, and then conducted sensitivity analyses by removing a study one-by-one to test whether the pooled results of remaining studies could be significantly changed (Egger et al., 1997). In contrast, fixed-effects models were employed. Potential publication bias was assessed using funnel plots when at least eight studies were available, supplemented by Egger’s test (*p* < 0.05 indicating potential bias). Statistical significance was set at *p* < 0.05.

## Results

### Literature search results

**Figure 1:**
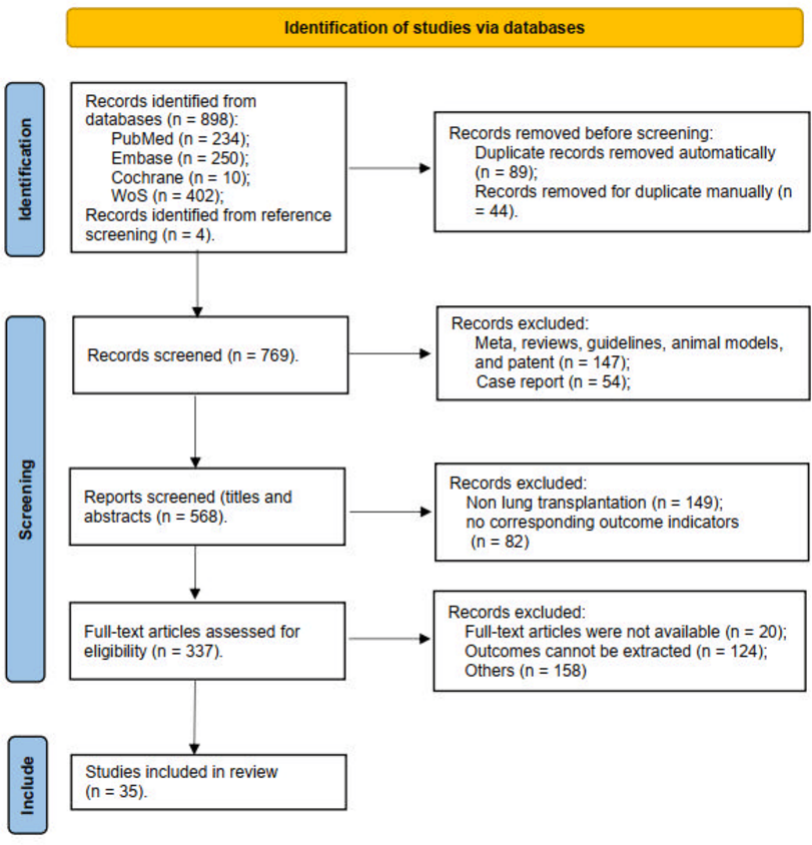
Flow chart of study identification, inclusion, and exclusion based on PRISMA guidelines.

Following the search strategy, a total of 896 records were initially identified from the four databases, with an additional four records recognized through reference list screening. After removing 129 duplicates automatically and manually, the remaining 769 records were screened, leading to the removal of 147 records categorized as meta-analyses, reviews, guidelines, or animal models, and 54 records with case reports. Subsequently, a review of the titles and abstracts of the remaining records led to the removal of 231 records based on the predefined criteria for inclusion and exclusion as previously described. Full-text articles were evaluated, and 302 records were excluded due to unavailability of full texts or lack of relevant outcomes. Ultimately, the present study incorporated a total of 35 studies. The detailed selection process is displayed in Fig. 1.

### Study characteristics

A total of 35 studies with 5,245 patients who underwent lung transplantation were included. Regarding geographic distribution, the majority of studies were conducted in the United States (*n* = 23), followed by Europe (*n* = 7), Canada (*n* = 1), New Zealand (*n* = 1), and Japan (*n* = 1). In terms of study design, 29 studies were retrospective cohort studies, while four were prospective cohort studies. The sample size of the included studies ranged from 24 to 457 patients, the proportion of male participants ranged from 33.3% to 75.8%, and the mean age of study populations ranged from 38.0 to 61.0 years. Regarding the diagnosis criteria for OPD, most studies employed modified barium swallow study (MBSS) and fiberoptic endoscopic evaluation of swallowing (FEES). Other assessment tools included the Penetration-Aspiration Scale and evaluation by speech-language pathologists. For the diagnosis of GERD, all but one study reporting its prevalence utilized pH monitoring, either alone or in combination with other diagnostic modalities such as endoscopy or manometry. The clinical characteristics of all included studies are shown in Table 1 (Atkins et al., 2010; Atkins et al., 2007; Barilka et al., 2025; Baumann et al., 2017; Bhat et al., 2021; Blondeau et al., 2007; Blondeau et al., 2009; Bobadilla et al., 2010; Cantu 3rd et al. 2004; Dallal-York et al., 2023; Dallal-York et al., 2022; Davis et al., 2010; Davis Jr et al., 2003; De Pablo et al., 2021; Gouynou et al., 2020; Graham et al., 2024a; Graham et al., 2024b; Grass et al., 2015; Green et al., 2023; Hamid et al., 2024; Kayawake et al., 2018; King et al., 2009; Lill et al., 2020; Lo et al., 2023; Lo et al., 2024; Masuda et al., 2018; Mendez et al., 2012; Miles et al., 2021; Molina et al., 2009; Neelankavil et al., 2024; Reedy et al., 2023; Shah et al., 2010; Skoretz et al., 2010; Soresi et al., 2016; Yang et al., 2023a).

**Table 1:** Basic characteristics of the included studies.

**Studies**	**Countries**	**Design**	**Data collect** **period**	**Samples** **(n)**	**Gender** **F (%)**	**Age** **(M)**	**Evaluation tool**	**Outcomes reported**
Atkins et al. (2010)	USA	Retrospective	2001–2005	263	75.81	49.00	FEES or VFSS	OPD/GERD rate, age, BSA
Graham et al. (2024a), Graham et al. (2024b)	USA	Retrospective	2018–2022	118	NA	NA	SLP, MBSS	OPD rate, age, BSA
Dallal-York et al. (2023)	USA	Prospective	2020–2021	45	73.00	60.10	FEES	OPD rate
Graham et al. (2024a), Graham et al. (2024b)	USA	Prospective	NA	50	58.00	59.64	MBSS	OPD rate
Barilka et al. (2025)	USA	Retrospective	2020–2024	392	59.00	54.00	MBSS	OPD rate
Baumann et al. (2017)	USA	Retrospective	2009–2012	321	55.60	56.20	FEES or MBSS	OPD rate
Reedy et al. (2023)	USA	Retrospective	2016–2021	42	NA	58.36	MBSS	OPD rate
Neelankavil et al. (2024)	USA	Retrospective	2020–2021	158	NA	61.00	FEES	OPD rate
Dallal-York et al. (2022)	USA	Retrospective	2017–2020	205	51.00	58.60	PAS	OPD rate
Atkins et al. (2007)	USA	Retrospective	2001–2002	263	NA	49.00	FEES	OPD rate
Miles et al. (2021b)	New Zealand	Retrospective	2014–2018	101	47.52	50.00	SLP	OPD rate
Skoretz et al. (2010)	Canada	Retrospective	2001–2004	69	NA	NA	VFSS	OPD rate
Lill et al. (2020)	USA	Retrospective	2012–2015	257	64.20	58.00	MBSS	OPD rate
Blondeau et al. (2007)	Belgium	Retrospective	1996–2005	45	35.60	52.00	24 h EI-pH	GERD rate
Blondeau et al. (2009)	Belgium	Retrospective	NA	24	37.50	52.00	24 h EI-pH	GERD rate
Bobadilla et al. (2010)	USA	Retrospective	1998–2007	54	38.50	48.00	Endoscopy, EI-pH, manometry	GERD rate, COPD, CF, IPF
Cantu 3rd et al. (2004)	USA	Retrospective	1992–2003	457	44.20	45.25	24 h EI-pH	GERD rate
Lo et al. (2023)	USA	Retrospective	2007–2016	175	59.00	56.30	24 h pH, MII-pH	GERD rate, CLAD, BOS, COPD, CF, IPF
Davis et al. (2010)	USA	Retrospective	NA	35	34.29	57.00	EHRIM, 24 h ADS-pH, endoscopy	GERD rate, COPD, CF, IPF, DGE
De Pablo et al. (2021)	Spain	Prospective	2008–2014	76	44.00	52.00	24H ambulatory-pH	GERD rate, survival
Yang et al. (2023a)	USA	Retrospective	2015–2016	76	69.70	60.00	MII-pH and HRM	GERD rate, CLAD, survival
Lo et al. (2024)	USA	Retrospective	NA	68	57.37	55.15	MII-pH and AET	GERD rate, CLAD, IPF
Gouynou et al. (2020)	France	Retrospective	2007–2017	93	56.00	43.30	HRM, EI-pH	GERD rate, CF
Grass et al. (2015)	Switzerland	Retrospective	1993–2010	205	NA	NA	pH, barium swallow and gastroscopy	GERD rate
Green et al. (2023)	USA	Retrospective	2006–2011	542	43.50	55.00	24 h EI-pH	GERD rate
Kayawake et al. (2018)	Japan	Retrospective	2008–2017	160	53.13	42.50	NA	GERD rate, CLAD, survival
Masuda et al. (2018)	USA	Prospective	2015–2016	77	57.14	64.00	HRM,24 h pH	GERD rate
Mendez et al. (2012)	USA	Retrospective	2008–2009	88	52.27	43.50	EHRIM, DS-pH	GERD rate, DGE
Molina et al. (2009)	USA	Retrospective	1994–2006	162	51.85	54.15	Endoscopy and/or esophagography	GERD rate, survival, BOS, COPD, IPF, DGE
Shah et al. (2010)	Georgia	Retrospective	2000–2009	69	49.27	58.00	pH probe manometry	GERD rate
Soresi et al. (2016)	Germany	Retrospective	2013–2014	84	50.00	48.00	pH, RAS	GERD rate, IPF
Bhat et al. (2021)	USA	Retrospective	2017–2020	69	33.30	63.90	Self-reported reflux	GERD rate
Hamid et al. (2024)	France	Retrospective	2001–2014	284	51.00	38.00	pH, DeMeester score > 14.7	GERD rate, CLAD
Davis Jr et al. (2023)	USA	Retrospective	1992–2002	128	35.15	49.00	pH	GERD rate
King et al. (2009)	UK	Retrospective	NA	59	–	48.50	Self-reported reflux	GERD rate, CLAD

**Notes.**

LT lung transplantation HT heart transplantation FEES fiberoptic endoscopic evaluation of swallowing VFSS videofluoroscopic swallowing study OPD oropharyngeal dysphagia GERD gastroesophageal reflux disease BSA body surface area SLP speech-language pathologist MBSS modified barium swallow study PAS Penetration-Aspiration Scale 24-h EI 24-hour esophageal impedance MII multichannel intraluminal impedance HRM high-resolution manometry AET acid exposure time NA no information DS dual-sensor RAS Reflux Symptom Index Questionnaire BOS bronchiolitis obliterans syndrome CF cystic fibrosis IPF idiopathic pulmonary fibrosis COPD chronic obstructive pulmonary disease DGE delayed gastric emptying CLAD chronic lung allograft dysfunction.

### Quality assessment of the included studies

Based on the NOS assessment, the methodological quality of the included studies was generally favorable, with an average score of 7.5 (range 6–9, Table 2). Among them, 29 studies were classified as having a low risk of bias (Atkins et al., 2010; Atkins et al., 2007; Barilka et al., 2025; Bhat et al., 2021; Bobadilla et al., 2010; Cantu 3rd et al. 2004; Dallal-York et al., 2023; Dallal-York et al., 2022; Davis Jr et al., 2003; De Pablo et al., 2021; Gouynou et al., 2020; Graham et al., 2024a; Graham et al., 2024b; Grass et al., 2015; Green et al., 2023; Hamid et al., 2024; King et al., 2009; Lill et al., 2020; Lo et al., 2023; Lo et al., 2024; Masuda et al., 2018; Miles et al., 2021; Molina et al., 2009; Neelankavil et al., 2024; Reedy et al., 2023; Shah et al., 2010; Skoretz et al., 2010; Soresi et al., 2016; Yang et al., 2023a), while six studies were rated as high risk (Baumann et al., 2017; Blondeau et al., 2007; Blondeau et al., 2009; Davis et al., 2010; Kayawake et al., 2018; Mendez et al., 2012). In detail, five studies achieved the maximum score of nine stars (Dallal-York et al., 2023; Gouynou et al., 2020; Green et al., 2023; Lo et al., 2023; Soresi et al., 2016), reflecting methodological rigor across all domains: selection, comparability, and outcome. The majority of studies scored between seven and eight stars, indicating generally sound methodology with minor limitations in areas such as cohort representativeness or confounding control. A smaller subset of studies (*e.g.*, Baumann et al., 2017; Blondeau et al., 2009; Davis et al., 2010; Kayawake et al., 2018; Mendez et al., 2012) scored six stars (Blondeau et al., 2007), indicating more prominent limitations, such as insufficient representativeness of the study population, inadequate adjustment for confounding factors, or less rigorous approaches to outcome measurement (like less precise outcome definitions or incomplete follow-up data). Overall, the body of evidence incorporated in this review demonstrates good to high quality, with the higher-rated studies contributing robust and reliable findings.

**Table 2:** Methodological quality of the included studies based on the Newcastle-Ottowa Scale.

**Studies**	**Study population selection**	**Comparability between group**	**Comparability between group**	**Total scores**
Atkins et al. (2010)	✩✩✩	✩✩	✩✩✩	8✩
Graham et al. (2024a), Graham et al. (2024b)	✩✩✩✩	✩✩	✩	7✩
Dallal-York et al. (2023)	✩✩✩✩	✩✩	✩✩✩	9✩
Graham et al. (2024a), Graham et al. (2024b)	✩✩✩	✩✩	✩✩	7✩
Barilka et al. (2025)	✩✩✩	✩✩	✩✩✩	8✩
Baumann et al. (2017)	✩✩✩	✩	✩✩	6✩
Reedy et al. (2023)	✩✩✩	✩	✩✩✩	7✩
Neelankavil et al. (2024)	✩✩✩✩	✩	✩✩	7✩
Dallal-York et al. (2022)	✩✩✩	✩✩	✩✩✩	8✩
Atkins et al. (2007)	✩✩✩✩	✩✩	✩✩	8✩
Miles et al. (2021b)	✩✩✩	✩✩	✩✩	7✩
Skoretz et al. (2010)	✩✩✩✩	✩✩	✩✩	8✩
Lill et al. (2020)	✩✩✩	✩✩	✩✩	7✩
Blondeau et al. (2007)	✩✩✩✩		✩✩✩	7✩
Blondeau et al. (2009)	✩✩✩	✩	✩✩	6✩
Bobadilla et al. (2010)	✩✩✩	✩✩	✩✩✩	8✩
Cantu 3rd et al. (2004)	✩✩✩✩	✩	✩✩	7✩
Lo et al. (2023)	✩✩✩✩	✩✩	✩✩✩	9✩
Davis et al. (2010)	✩✩✩	✩✩	✩	6✩
De Pablo et al. (2021)	✩✩✩✩	✩✩	✩✩	8✩
Yang et al. (2023a)	✩✩✩	✩✩	✩✩	7✩
Lo et al. (2024)	✩✩✩✩	✩	✩✩	7✩
Gouynou et al. (2020)	✩✩✩✩	✩✩	✩✩✩	9✩
Grass et al. (2015)	✩✩✩✩	✩✩	✩✩	8✩
Green et al. (2023)	✩✩✩✩	✩✩	✩✩✩	9✩
Kayawake et al. (2018)	✩✩✩	✩	✩✩	6✩
Masuda et al. (2018)	✩✩✩	✩✩	✩✩✩	8✩
Mendez et al. (2012)	✩✩✩	✩	✩✩	6✩
Molina et al. (2009)	✩✩✩✩	✩	✩✩	7✩
Shah et al. (2010)	✩✩	✩✩	✩✩	8✩
Soresi et al. (2016)	✩✩✩✩	✩✩	✩✩✩	9✩
Bhat et al. (2021)	✩✩✩	✩✩	✩✩	7✩
Hamid et al. (2024)	✩✩✩✩	✩✩	✩✩	8✩
Davis Jr et al. (2023)	✩✩✩	✩✩	✩✩	7✩
King et al. (2009)	✩✩✩✩	✩	✩✩	7✩

### Meta-analysis results

#### Prevalence of OPD in patients after lung transplantation

**Figure 2:**
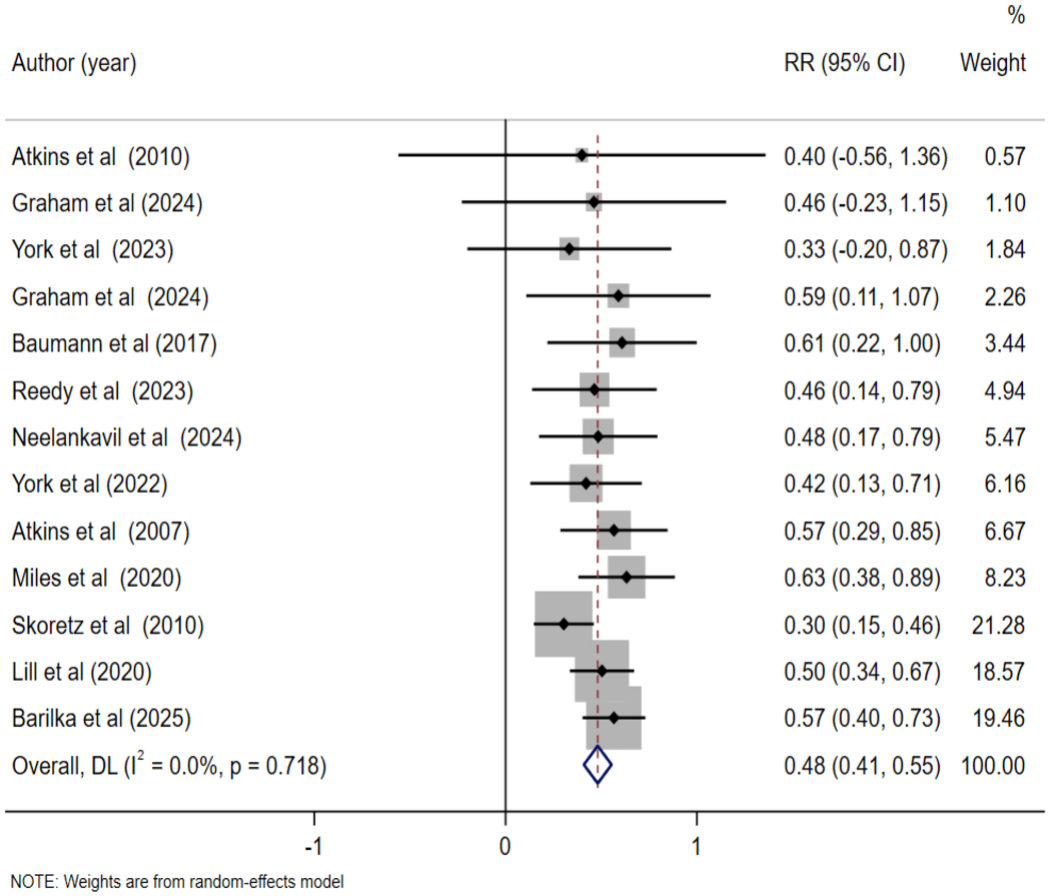
Prevalence of oropharyngeal dysphagia in patients after lung transplantation.

A total of 13 studies reported the prevalence of OPD (Atkins et al., 2010; Atkins et al., 2007; Barilka et al., 2025; Baumann et al., 2017; Dallal-York et al., 2023; Dallal-York et al., 2022; Graham et al., 2024a; Graham et al., 2024b; Lill et al., 2020; Miles et al., 2021; Neelankavil et al., 2024; Reedy et al., 2023; Skoretz et al., 2010). A fixed-effects model was applied for meta-analysis given the absence of significant heterogeneity among studies (*I*^2^ = 0.0%, *p* = 0.718). The results of meta-analyses showed that the prevalence of OPD was 48% (95% CI [0.41–0.55]), (Fig. 2). Despite the considerable heterogeneity observed, sensitivity analysis (Fig. 1S) did not reveal any single study as the primary source of variation. Subgroup analyses were conducted based on clinical features of OPD (*e.g.*, airway invasion, delayed swallow initiation, aspiration, laryngeal penetration or aspiration, and penetration), patient age (mean age ≤55 years *vs.* >55 years), and study design (retrospective *vs.* prospective). The results showed that the highest prevalence of OPD was observed for delayed swallow initiation (74%, 95% CI [0.12–1.35]), followed by penetration (58%, 95% CI [0.25–0.91]), while laryngeal penetration or aspiration had the lowest prevalence (24%, 95% CI [0.02–0.45]). Patients with a mean age ≤55 years had a higher OPD prevalence (58%, 95% CI (0.46, 0.70)) compared to those >55 years (49%, 95% CI [0.38–0.60]). No significant difference was found between retrospective and prospective studies. These findings suggest that OPD clinical features, patient age, and study design may represent potential sources of heterogeneity in the reported prevalence estimates. No evidence of publication bias was detected, as indicated by funnel plot symmetry (Fig. 2S) and a non-significant Egger’s test result (*p* = 0.373).

### Prevalence of GERD in patients after lung transplantation

**Figure 3:**
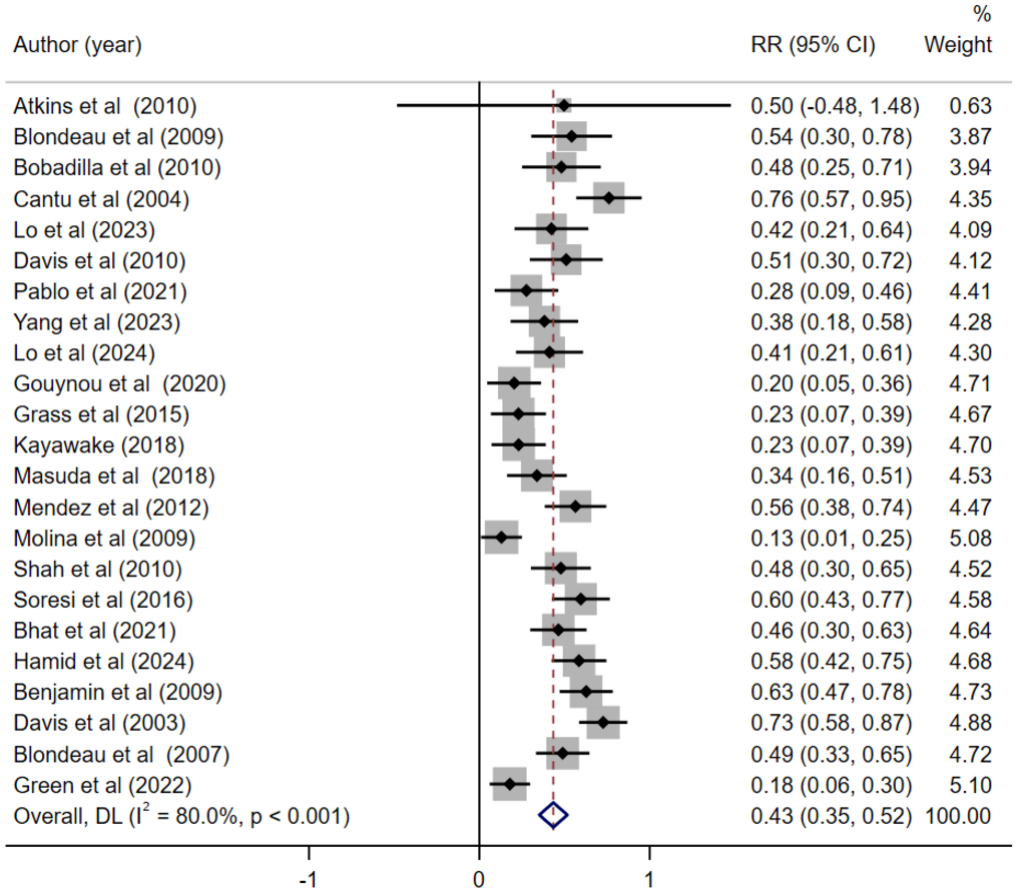
Prevalence of gastroesophageal reflux disease in patients after lung transplantation.

**Figure 4:**
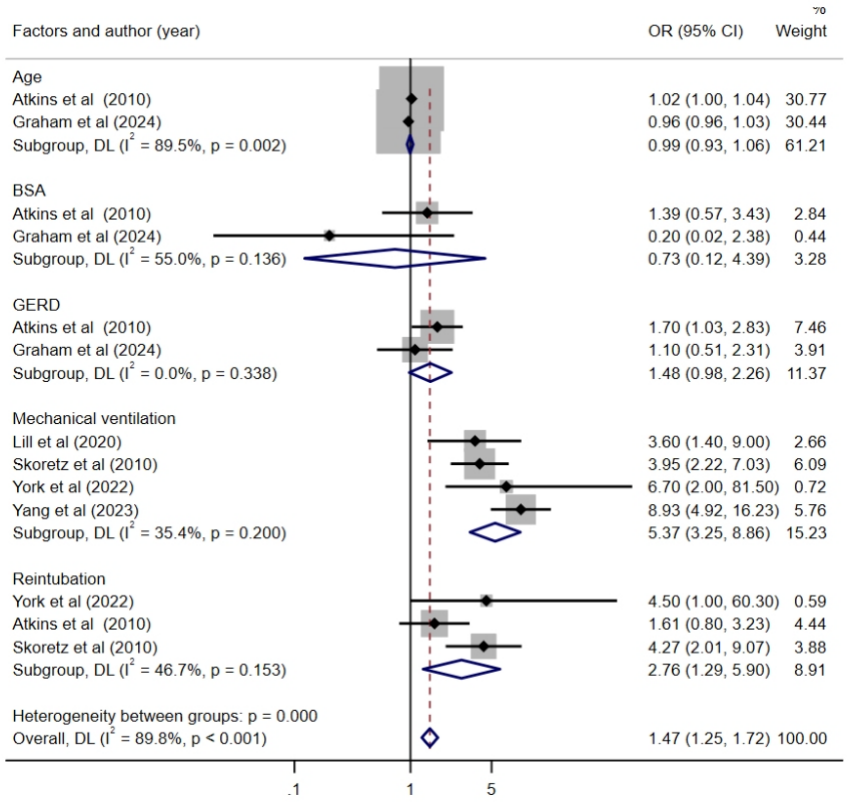
Factors for the risk of oropharyngeal dysphagia in patients after lung transplantation.

A total of 23 studies involving 2,776 patients reported the prevalence of GERD in patients after lung transplantation (Atkins et al., 2010; Bhat et al., 2021; Blondeau et al., 2007; Blondeau et al., 2009; Bobadilla et al., 2010; Cantu 3rd et al. 2004; Davis et al., 2010; Davis Jr et al., 2003; De Pablo et al., 2021; Gouynou et al., 2020; Grass et al., 2015; Green et al., 2023; Hamid et al., 2024; Kayawake et al., 2018; King et al., 2009; Lo et al., 2023; Lo et al., 2024; Masuda et al., 2018; Mendez et al., 2012; Molina et al., 2009; Shah et al., 2010; Soresi et al., 2016; Yang et al., 2023a). Due to substantial heterogeneity among the included studies (*I*^2^ = 80.0%, *p* < 0.001, Fig. 3), a random-effects model was used for meta-analysis. The pooled prevalence of GERD was 43% (95% CI [0.35–0.52]). Despite the considerable heterogeneity observed, sensitivity analysis (Fig. 3S) did not reveal any single study as the primary source of variation. Subgroup analyses were performed to examine potential sources of heterogeneity, categorizing studies by diagnostic criteria (only used pH monitoring, pH monitoring and other tools such as endoscopy and/or manometry, and other tools such as self-reported reflux), patient age (mean age ≤55 years *vs.* >55 years), and study design (retrospective *vs.* prospective). These analyses revealed that patient age and study design were contributors to heterogeneity, whereas diagnostic criteria was not (Table 3). Specifically, the use of pH monitoring alone was associated with a higher pooled GERD prevalence (60%, 95% CI [0.36–0.73]) compared to combination with other tools (42%, 95% CI [0.34–0.50]). Similarly, retrospective studies showed a higher prevalence (45%, 95% CI [0.31–0.55]) than prospective studies (31%, 95% CI [0.18–0.44]). Similar prevalence of GERD between studies with a mean patient age >55 years and those with a mean age ≤55 years (Table 4). No significant publication bias was detected, as indicated by funnel plot inspection (Fig. 4S) and Egger’s test (*p* = 0.153).

**Table 3:** Subgroup analyses of the prevalence of oropharyngeal dysphagia among the included studies.

**Subgroup analyses**	**Numbers of the** **included studies**	**OPD prevalence**		**Heterogeneity**	
		**RR (95% CI)**	***p***	***I*** ^**2**^	***p***
**Evaluation tools**					
OPD	6	0.33 (0.22, 0.43)	*<0.001*	23.1%	*0.261*
Airway invasion	3	0.54 (0.12, 0.96)	*<0.001*	64.4%	*0.060*
Delayed swallow initiation	2	0.74 (0.12, 1.35)	*<0.001*	72.6%	*0.056*
Aspiration	6	0.59 (0.47, 0.71)	*<0.001*	0.0%	*0.571*
Laryngeal penetration or aspiration	2	0.24 (0.02, 0.45)	*<0.001*	5.2%	*0.304*
Penetration	2	0.58 (0.25, 0.91)	*<0.001*	76.2%	*0.040*
**Patient age**					
≤55	4	0.58 (0.46, 0.70)	*<0.001*	0.0%	*0.953*
>55	7	0.49 (0.38, 0.60)	*<0.001*	0.0%	*0.981*
**Study design**					
Retrospective	11	0.48 (0.41, 0.56)	*<0.001*	0.0%	*0.596*
Prospective	2	0.47 (0.12, 0.83)	*<0.001*	0.0%	*0.484*

**Table 4:** Subgroup analyses of the prevalence of gastroesophageal reflux disease among the included studies.

**Subgroup analyses**	**Numbers of the** **included studies**	**OPD prevalence**		**Heterogeneity**	
		**RR (95% CI)**	***p***	***I*** ^**2**^	***p***
**Evaluation tools**					
Only pH	6	0.50 (0.31, 0.69)	*<0.001*	86.1%	*<0.001*
pH and other evaluations	12	0.42 (0.34, 0.50)	*<0.001*	56.2%	*0.011*
Others	5	0.39 (0.13, 0.66)	*<0.001*	90.9%	*<0.001*
**Age**					
≤55	14	0.43 (0.31, 0.55)	*<0.001*	85.5%	*<0.001*
>55	8	0.47 (0.38, 0.56)	*<0.001*	43.6%	*0.088*
**Study design**					
Retrospective	21	0.45 (0.36, 0.54)	*<0.001*	81.3%	*<0.001*
Prospective	2	0.31 (0.18, 0.44)	*<0.001*	0.0%	*0.639*

### Factors associated with the risk of OPD

A total of six studies reported risk factors of OPD (Atkins et al., 2010; Dallal-York et al., 2022; Graham et al., 2024a; Lill et al., 2020; Skoretz et al., 2010; Yang et al., 2023a). Among these, five factors were included in meta-analyses: age (Atkins et al., 2010; Graham et al., 2024a), body surface area (BSA) (Atkins et al., 2010; Graham et al., 2024a), GERD (Atkins et al., 2010; Graham et al., 2024a), mechanical ventilation (Dallal-York et al., 2022; Lill et al., 2020; Skoretz et al., 2010; Yang et al., 2023a), and reintubation (Atkins et al., 2010; Dallal-York et al., 2022; Skoretz et al., 2010). Due to substantial heterogeneity across studies (*I*^2^ = 89.8%, *p* < 0.001), random-effects models were applied. Meta-analysis revealed that mechanical ventilation (Fig. 4, OR = 5.37, 95% CI [3.25–8.86]), *p* = 0.009) and reintubation (OR = 2.76, 95% CI [1.29–5.90], *p* < 0.009) were significant risk factors for OPD, while age, BSA, and GERD did not reach significance (age, OR = 0.99, 95% CI [0.93–1.04], *p* = 0.806; BSA, OR = 0.73, 95% CI [0.12–4.39], *p* = 0.734; GERD, OR = 1.48, 95% CI [0.98–2.26], *p* = 0.065). Considerable heterogeneity was observed in the analysis of age as a risk factor (*I*^2^ = 89.5%, *p* = 0.002). No significant publication bias was detected for studies evaluating mechanical ventilation (Egger’s test *p* = 0.920) or reintubation (Egger’s test *p* = 0.767).

### The incidence of clinical events between GERD patients and non-GERD patients

**Figure 5:**
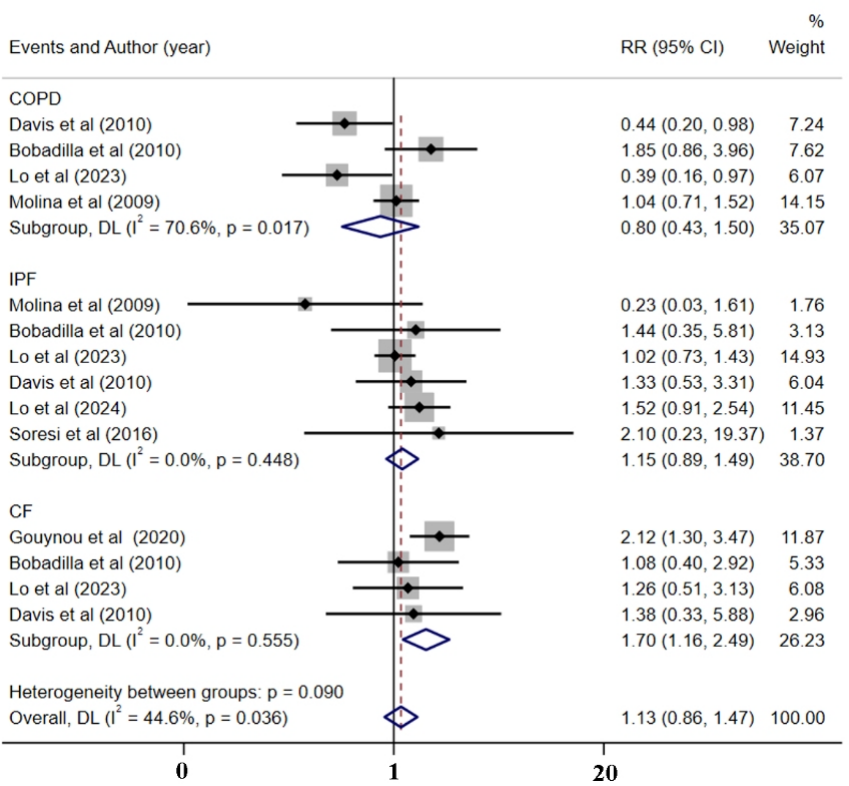
The incidence of clinical events between GERD patients and non-GERD patients. COPD, chronic obstructive pulmonary disease; IPF, idiopathic pulmonary fibrosis; CF, cystic fibrosis.

Among the included studies, four studies assessed the incidence of COPD between GERD patients and non-GERD patients (Bobadilla et al., 2010; Davis et al., 2010; Lo et al., 2023; Molina et al., 2009), six assessed IPF incidence (Bobadilla et al., 2010; Davis et al., 2010; Lo et al., 2023; Lo et al., 2024; Molina et al., 2009; Soresi et al., 2016), and four studies assessed CF incidence (Bobadilla et al., 2010; Davis et al., 2010; Gouynou et al., 2020; Lo et al., 2023). Meta-analysis (Fig. 5) demonstrated a significantly higher incidence of CF in GERD patients compared to non-GERD patients (RR = 1.70, 95% CI [1.16–2.49], *p* = 0.006). In contrast, no significant differences were observed in the incidence of COPD (RR = 0.80, 95% CI [0.43–1.50], *p* = 0.488) or IPF between GERD patients and non-GERD patients (RR = 1.15, 95% CI [0.89–1.49], *p* = 0.294). Substantial heterogeneity was noted in the COPD analysis (*I*^2^ = 70.6%, *p* = 0.017, Fig. 5), though sensitivity analysis did not identify its source (Fig. 5S). Egger’s tests revealed no significant publication bias for any of the outcomes (COPD, *p* = 0.541; IPF, *p* = 0.951; CF, *p* = 0.154).

### The predictive value of GERD on clinical outcomes in patients after lung transplantation.

**Figure 6:**
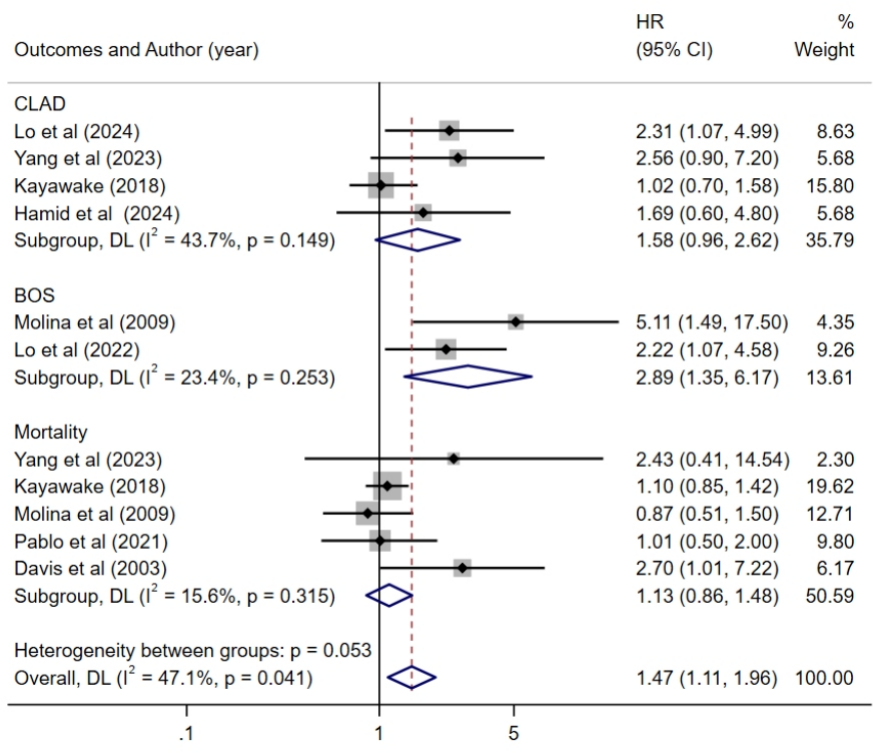
Factors for the risk of gastroesophageal reflux disease in patients after lung transplantation.

Seven studies investigated the predictive value of GERD on clinical outcomes (Davis Jr et al., 2003; De Pablo et al., 2021; (Hamid et al., 2024; Kayawake et al., 2018; Lo et al., 2024; Molina et al., 2009; Yang et al., 2023a), with four reporting on chronic lung allograft dysfunction (CLAD) (Hamid et al., 2024; Kayawake et al., 2018; Lo et al., 2024; Yang et al., 2023a), two on bronchiolitis obliterans syndrome (BOS) (Lo et al., 2023; Molina et al., 2009), and five on mortality (Davis Jr et al., 2003; De Pablo et al., 2021; (Kayawake et al., 2018; Molina et al., 2009; Yang et al., 2023a). Meta-analysis demonstrated that GERD was a significant predictor for BOS (HR = 2.89, 95% CI [1.35–6.17], *p* = 0.006), while was not for CLAD (HR = 1.58, 95% CI [0.96–2.62], *p* = 0.074) and mortality (HR = 1.13, 95% CI [0.86–1.48], *p* = 0.398, Fig. 6). Egger’s test suggested no potential publication bias for CLAD studies (*p* = 0.079) and mortality (*p* = 0.760).

## Discussion

This systematic review and meta-analysis provide a quantitative synthesis of the existing literature, to our knowledge the most comprehensive to date, quantifying the prevalence of OPD and GERD, identifying risk factors for OPD, and assessing the impact of GERD on clinical outcomes in lung transplant patients. The key findings demonstrate that both disorders are highly prevalent in this patient population, with a combined prevalence of 48% for OPD and 43% for GERD. Several clinically significant risk factors for OPD were identified, including mechanical ventilation and reintubation. Furthermore, CF occurred significantly more frequently in patients with GERD compared to those without the disease. Additionally, GERD was identified as a critical risk factor for BOS, reflecting its role in exacerbating chronic allograft dysfunction.

The current meta-analysis found a pooled OPD prevalence of 48% following lung transplantation, which is markedly higher than the estimated prevalence in the general community-dwelling adult population (approximately 8–15%, varying by age and diagnostic criteria) (Yang et al., 2023b). This figure is consistent with the 43.8% global prevalence reported in a prior systematic review that included multiple at-risk populations, such as individuals with stroke, head and neck cancer, and neurodegenerative diseases (Rajati et al., 2022). These findings indicate that lung transplant recipients experience OPD at rates comparable to those of other well-established high-risk groups, underscoring the clinical relevance of dysphagia in this population. OPD, most commonly represents the leading cause of late morbidity and mortality following lung transplantation. The pathogenesis of OPD after lung transplantation is complex and multifactorial, involving intraoperative injury to the vagus and recurrent laryngeal nerves during pulmonary artery dissection (Altman, Yu & Schaefer, 2010; Jadcherla, Litzenberg & Balasubramanian, 2023), which may impair laryngeal sensation and motor function, together with iatrogenic factors such as postoperative tracheostomy, prolonged mechanical ventilation, enteral feeding tubes, and repeated endoscopic procedures (Jadcherla, Litzenberg & Balasubramanian, 2023; Rommel & Hamdy, 2016; Skoretz et al., 2020). Subgroup analysis demonstrated that younger patients (aged ≤55 years) were more likely to develop OPD. This finding contrasts with prior research that suggested a higher prevalence of dysphagia in older populations (McCoy & Desai, 2018; Zhang et al., 2021b). This discrepancy may be explained by the limited number of studies in older age groups included in our analysis (only three studies), as well as differences in age stratification (our study used a cut-off of 55 years, whereas previous studies used 75 years). Importantly, advanced age is a relative contraindication for lung transplantation, with many centers applying upper age limits for transplant candidacy. Consequently, the lung transplant population is inherently selected for younger, healthier older adults, which may attenuate the expected age-related increase in dysphagia risk. Lung transplantation represents a unique context for dysphagia, which has been associated with various severe clinical complications, including aspiration, reflux, pharyngeal residue, excessive secretions, malnutrition, dehydration, prolonged hospital stay, and mortality. The underlying pathophysiology in this population involves multiple mechanisms, including potential intraoperative injury to the vagus and recurrent laryngeal nerves during pulmonary artery dissection (Altman, Yu & Schaefer, 2010; Jadcherla, Litzenberg & Balasubramanian, 2023), which may result in impaired laryngeal sensation and motor dysfunction. Additionally, several iatrogenic factors contribute to dysphagia, including postoperative tracheostomy, prolonged mechanical ventilation, use of enteral feeding tubes, and repeated endoscopic procedures(Jadcherla, Litzenberg & Balasubramanian, 2023; Rommel & Hamdy, 2016; Skoretz et al., 2020). Importantly, emerging evidence suggests that these complications are modifiable. A structured three-month dysphagia intervention program incorporating swallowing therapy and respiratory coordination has been shown to significantly reduce pneumonia rates in this at-risk population (Black et al., 2020). These findings underscore the necessity of establishing a systematic screening protocol for dysphagia and conducting thorough risk factor assessments during the early post-transplant period. To effectively mitigate complications and improve overall clinical outcomes, implementation of a multidisciplinary team approach involving speech-language pathologists, pulmonologists, transplant surgeons, and rehabilitation specialists is strongly recommended. This team should focus on early integrated swallowing and respiratory rehabilitation as a standard component of postoperative care.

Mechanical ventilation and reintubation were identified as significant risk factors for OPD in our analysis, consistent with previous studies conducted in intensive care units (Schefold et al., 2017; Zuercher et al., 2020), neurocritical care units (Reitz et al., 2024), and post-heart transplantation settings (Plowman et al., 2023). Although the pathophysiology of dysphagia in lung transplant recipients is multifactorial and not fully elucidated, endotracheal intubation and prolonged mechanical ventilation are likely contributing factors. The pathophysiological mechanisms involve structural, neurological, and functional interactions. Structurally, endotracheal intubation may cause trauma to laryngeal and pharyngeal tissues, resulting in edema, ulceration, or vocal cord impairment (Zuercher et al., 2019). Neurologically, prolonged presence of the endotracheal tube exerts continuous pressure on laryngeal structures, compressing both mucosal and neural tissues, which may compromise the superior and recurrent laryngeal nerves (Vahabzadeh-Hagh et al., 2023). This neural impairment leads to diminished laryngeal sensation and impaired vocal fold adduction, both of which are essential for airway protection during swallowing. Physiologically, prolonged ventilation results in disuse atrophy of oropharyngeal muscles, and the process of weaning and extubation does not guarantee immediate recovery of finely coordinated swallowing reflexes (Beuret et al., 2024; Likar et al., 2024). Reintubation further exacerbates these effects, often occurring under emergent circumstances that increase the risk of additional trauma (Taboada et al., 2021). Collectively, these factors (sensory impairment, muscular weakness, and structural injury) disrupt the sophisticated coordination required for safe swallowing, thereby elevating the risk of aspiration and clinically significant dysphagia (Hoh & Semrau, 2025). Based on our findings, we recommend that preventive strategies focus on modifiable risk factors, especially mechanical ventilation and reintubation. Early extubation protocols, careful airway management to minimize laryngeal trauma, and systematic assessment of swallowing function prior to oral feeding should be implemented. For patients requiring prolonged ventilation, early involvement of speech-language pathologists for swallowing evaluation and rehabilitation is essential. A structured, multidisciplinary approach involving pulmonologists, transplant surgeons, speech-language pathologists, and rehabilitation specialists should be established to implement early swallowing and respiratory rehabilitation as a standard component of postoperative care. However, the current study did not found the significant association between GERD and OPD. Current clinical practice regarding the routine assessment of OPD and GERD varies considerably across transplant centers. For OPD, standardized screening protocols are not universally implemented, despite the high prevalence and associated morbidity identified in our analysis. For GERD, while the International Society for Heart and Lung Transplantation (ISHLT) guidelines recommend pre-transplant evaluation, post-transplant surveillance protocols vary; some centers perform routine pH monitoring or impedance testing within the first year, while others adopt a selective approach based on symptoms or pulmonary function decline (Peled et al., 2024; Velleca et al., 2023). Our findings underscore the need for more systematic, protocol-driven screening for both OPD and GERD in the post-transplant period, given their high prevalence and significant impact on graft outcomes. We suggest that routine post-transplant assessment should include objective swallowing evaluation and GERD testing (*e.g.*, pH monitoring) to enable early intervention and potentially mitigate the risk of chronic allograft dysfunction.

The pooled prevalence of GERD following lung transplantation was 41%, confirming its status as a major comorbidity in this population. This represents a substantial increase compared to the 13% prevalence of reflux symptoms reported in the general population (Nirwan et al., 2020) and aligns with previous studies that have documented a significant rise in reflux symptoms during the post-transplant period, ranging from 35% to 69% (Masuda et al., 2018; Young et al., 2003). The high prevalence of GERD carries important clinical implications, particularly regarding its association with long-term allograft dysfunction. GERD may contribute to non-immunologic allograft injury through aspiration of gastroesophageal contents, potentially leading to BOS (Patti et al., 2016). The mechanisms underlying increased GERD after lung transplantation remain controversial but likely involve a confluence of surgical, neuropathic, and pharmacological factors. Surgical dissection around the esophagus and lung implantation may compromise lower esophageal sphincter (LES) integrity and function (Tack & Pandolfino, 2018). Intraoperative vagus nerve injury can produce a dual pathology by impairing LES tone and causing gastroparesis, both of which promote reflux (Xiaoli et al., 2017; Zhang et al., 2021a). Immunosuppressants, particularly calcineurin inhibitors, may further exacerbate these mechanisms by contributing to decreased LES pressure (Mertens et al., 2009; Razia et al., 2023; Snyder & Katzka, 2022). This pathophysiology establishes a vicious cycle in which refluxed gastric contents damage the allograft, promoting chronic rejection while simultaneously worsening GERD symptoms. Our findings also demonstrated a higher incidence of CF in patients with GERD compared to those without GERD. Patients with CF are predisposed to GERD through a combination of anatomical, neuromuscular, and physiological abnormalities (Bongiovanni et al., 2020; Maqbool & Pauwels, 2017). Gastric emptying may be delayed and intra-abdominal pressure increased due to thickened secretions and pancreatic insufficiency, mechanically disrupting the esophagogastric junction through changes in pressure gradients and diaphragmatic flattening. Additionally, CF-related dysmotility extends to the esophagus, where reduced peristaltic efficiency and impaired acid clearance may exacerbate reflux (Dorsey & Gonska, 2017). This complex dysfunction enables both acid and non-acid reflux, creating a self-perpetuating cycle in which refluxate-induced bronchospasm worsens pulmonary function, and progressive lung disease further promotes reflux.

A significant association between GERD and the development of BOS following lung transplantation was identified in the current meta-analysis, which was consistent with previous reviews (Garrity Jr, 2013; Krahelski et al., 2025). BOS, characterized by progressive obstructive ventilatory defect resulting from obliterative bronchiolitis, is widely recognized as the greatest threat to long-term allograft function (Barker et al., 2014; Krishna, Anjum & Oliver, 2025; Verleden et al., 2019). Although a definitive causal relationship has not been established, numerous studies have proposed that GERD contributes to BOS development through silent aspiration of gastric contents, leading to direct airway injury and/or upregulation of pulmonary inflammatory responses (Blondeau et al., 2009; D’Ovidio et al., 2005; Stovold et al., 2007). This association may be primarily driven by chronic microaspiration of gastric and duodenal contents into the vulnerable lung allograft (Wood, 2015). In this mechanism, aspirated material is not chemically inert but contains a complex mixture of gastric and duodenal constituents, including not only acid but also pepsin, bile acids, and other non-acid refluxate, which induce direct chemical injury to the bronchial epithelium. Importantly, the aspirated material is also non-sterile, carrying oropharyngeal and gastric microbiota. Bacterial colonisation and repeated microaspiration of viable microorganisms introduce an additional microbiological challenge, leading to chronic airway infection, dysbiosis, and enhanced local inflammation. Together, chemical and microbial insults initiate a localised pro-inflammatory cascade, recruiting neutrophils and increasing pro-inflammatory cytokines such as interleukin-8 (IL-8), thereby creating a milieu that promotes CD8+ T-cell-mediated inflammation and subsequent fibrotic response (Fisichella et al., 2013; Hathorn, Chan & Lo, 2017). This persistent, combined chemical and infectious injury leads to the characteristic histopathological changes of fibrous scarring and small airway obliteration that define BOS. However, the observed association between GERD and BOS was derived from only two studies, limiting both the precision and generalizability of this finding. While the consistency of the effect direction across these studies supports the biological plausibility of this relationship, larger prospective cohort studies are needed to confirm this association and to evaluate the potential benefit of antireflux interventions on BOS prevention.

This study has several important limitations. First, substantial heterogeneity was observed in several pooled estimates, particularly for GERD prevalence and certain OPD risk factors. Although subgroup analyses were performed to explore potential sources of heterogeneity, significant variation persisted, likely attributable to methodological differences in diagnostic criteria across studies. This reflects a critical lack of standardization in defining these conditions within the literature and underscores the need for consistent diagnostic approaches in future research. Second, despite our systematic search strategy, We acknowledge that this may narrow the scope of our review regarding the comprehensive pathophysiology of GERD in lung transplant recipients, particularly the potential benefits of surgical antireflux interventions. Third, assessment for small-study effects using funnel plot inspection and Egger’s test was constrained by the limited number of studies available for certain comparisons; therefore, these results should be interpreted cautiously. Fourth, the observed association between GERD and BOS was derived from only two studies, limiting both the precision and generalizability of this finding and raising concerns regarding potential sampling bias. Fifth, despite our comprehensive search strategy across four major databases and manual screening of reference lists, we acknowledge that studies not indexed in the searched databases or with non-English titles/abstracts may have been missed. Future updates of this meta-analysis should consider broader search strategies including additional databases (*e.g.*, Scopus, CINAHL) and direct contact with study authors to identify unpublished longitudinal data. Notwithstanding these limitations, the consistently elevated prevalence rates reinforce that OPD and GERD represent significant comorbidities following lung transplantation and warrant greater clinical attention. The identification of modifiable factors such as mechanical ventilation offers opportunities to incorporate preventive strategies into perioperative care. The association between GERD and BOS, although based on limited data, reaffirms the role of reflux in long-term allograft dysfunction and emphasizes the importance of proactive reflux management in this patient population.

## Conclusion

In conclusion, this systematic review and meta-analysis demonstrates that OPD and GERD are highly prevalent and clinically significant comorbidities in the post-lung transplant population, with pooled prevalence rates of 48% and 43%, respectively. The particular and changeable risk factors of OPD, including mechanical ventilation and reintubation, are found in the list of our results, which provide a tangible goal of the prophylactic perioperative care. More importantly, our results reveal a strong correlation between GERD and its subsequent progression into the BOS, which contributes to its data as the major cause of chronic allograft impairment. All of these findings accentuate the necessity to incorporate a systematized, proactive screening regimen with regard to both OPD and GERD as part of the routine post-transplant care algorithms. Future research should not only implement and validate screening protocols and targeted interventions for OPD and GERD in lung transplant recipients but also prioritize systematic reviews of surgical (fundoplication) and longitudinal GERD studies to better clarify the temporal relationship between reflux and CLAD. These efforts are essential for preserving allograft function and improving long-term patient survival.

## Supplemental Information

10.7717/peerj.21472/supp-1 Supplemental Information 1 PRISMA checklist.

10.7717/peerj.21472/supp-2 Supplemental Information 2 Search strategy.

10.7717/peerj.21472/supp-3 Supplemental Information 3 Sensitivity analyses and funnel plots.

10.7717/peerj.21472/supp-4 Supplemental Information 4 Rationale for conducting the meta-Analysis and contribution to knowledge in light of previously published reports

10.7717/peerj.21472/supp-5 Supplemental Information 5 Raw data extracted from the cited literature for review and publication

## Additional Information and Declarations

### Data Availability

The following information was supplied regarding data availability: This is a Systematic review/Meta-analysis.
