# Supplementary material for: Oropharyngeal dysphagia and gastroesophageal reflux disease in lung transplant patients: a systematic review and meta-analysis of incidence, risk factors, and clinical outcomes
Source: PeerJ. 2026 Jul 6;14:e21472. doi: 10.7717/peerj.21472 (PMC13348482; doi:10.7717/peerj.21472)
Supplement: Supplemental Information 2 [file peerj-14-21472-s002.docx]

**Table 2S.** Literature search strategy of PubMed

| Search number | Query | Records |
| --- | --- | --- |
| #1 | "Lung Transplantation"[Mesh] | 20,524 |
| #2 | (((((((lung transplantation[Title/Abstract]) OR (Lung Grafting[Title/Abstract])) OR (Lung Graftings[Title/Abstract])) OR (Lung Transplantations[Title/Abstract])) OR (lung allotransplantation[Title/Abstract])) OR (lung orthotopic transplantation[Title/Abstract])) OR (lung tissue transplantation[Title/Abstract])) OR (pulmonary transplantation[Title/Abstract]) | 20,111 |
| #3 | #1 OR #2 | 27,006 |
| #4 | "Deglutition Disorders"[Mesh] | 64,696 |
| #5 | ((((((((((((((((((((Deglutition Disorders[Title/Abstract]) OR (Deglutition Disorder[Title/Abstract])) OR (Swallowing Disorders[Title/Abstract])) OR (Swallowing Disorder[Title/Abstract])) OR (Oropharyngeal Dysphagia[Title/Abstract])) OR (Esophageal Dysphagia[Title/Abstract])) OR (Aphagopraxia[Title/Abstract])) OR (deglutition difficulty[Title/Abstract])) OR (deglutition disorder[Title/Abstract])) OR (deglutition disorders[Title/Abstract])) OR (difficult deglutition[Title/Abstract])) OR (difficulty in swallowing[Title/Abstract])) OR (difficulty swallowing[Title/Abstract])) OR (dysphagias[Title/Abstract])) OR (swallowing difficult[Title/Abstract])) OR (swallowing difficultness[Title/Abstract])) OR (swallowing difficulty[Title/Abstract])) OR (swallowing disorder[Title/Abstract])) OR (Barium swallow[Title/Abstract])) OR (Flexible endoscopic evaluation of swallow[Title/Abstract])) OR (Videofuoroscopy[Title/Abstract]) | 10,522 |
| #6 | #4 OR #5 | 69,523 |
| #7 | "Gastroesophageal Reflux"[Mesh] | 31,271 |
| #8 | ((((((((((((((Gastroesophageal Reflux[Title/Abstract])) OR (Esophageal Reflux[Title/Abstract])) OR (Gastric Acid Reflux[Title/Abstract])) OR (Gastro-Esophageal Reflux[Title/Abstract])) OR (Gastro Esophageal Reflux[Title/Abstract])) OR (Gastro-oesophageal Reflux[Title/Abstract])) OR (Gastro oesophageal Reflux[Title/Abstract])) OR (Gastroesophageal Reflux Disease[Title/Abstract])) OR (GERD[Title/Abstract])) OR (Gastro-Esophageal Reflux Disease[Title/Abstract])) OR (Gastro Esophageal Reflux Disease[Title/Abstract])) OR (Gastro-Esophageal Reflux Diseases[Title/Abstract])) | 33,720 |
| #9 | #7 OR #8 | 44, 205 |
| #10 | #6 OR #9 | 81,523 |
| #11 | #3 AND #10 | 206 |
