# Supplementary material for: Oropharyngeal dysphagia and gastroesophageal reflux disease in lung transplant patients: a systematic review and meta-analysis of incidence, risk factors, and clinical outcomes
Source: PeerJ. 2026 Jul 6;14:e21472. doi: 10.7717/peerj.21472 (PMC13348482; doi:10.7717/peerj.21472)
Supplement: Supplemental Information 3 [file peerj-14-21472-s003.docx]

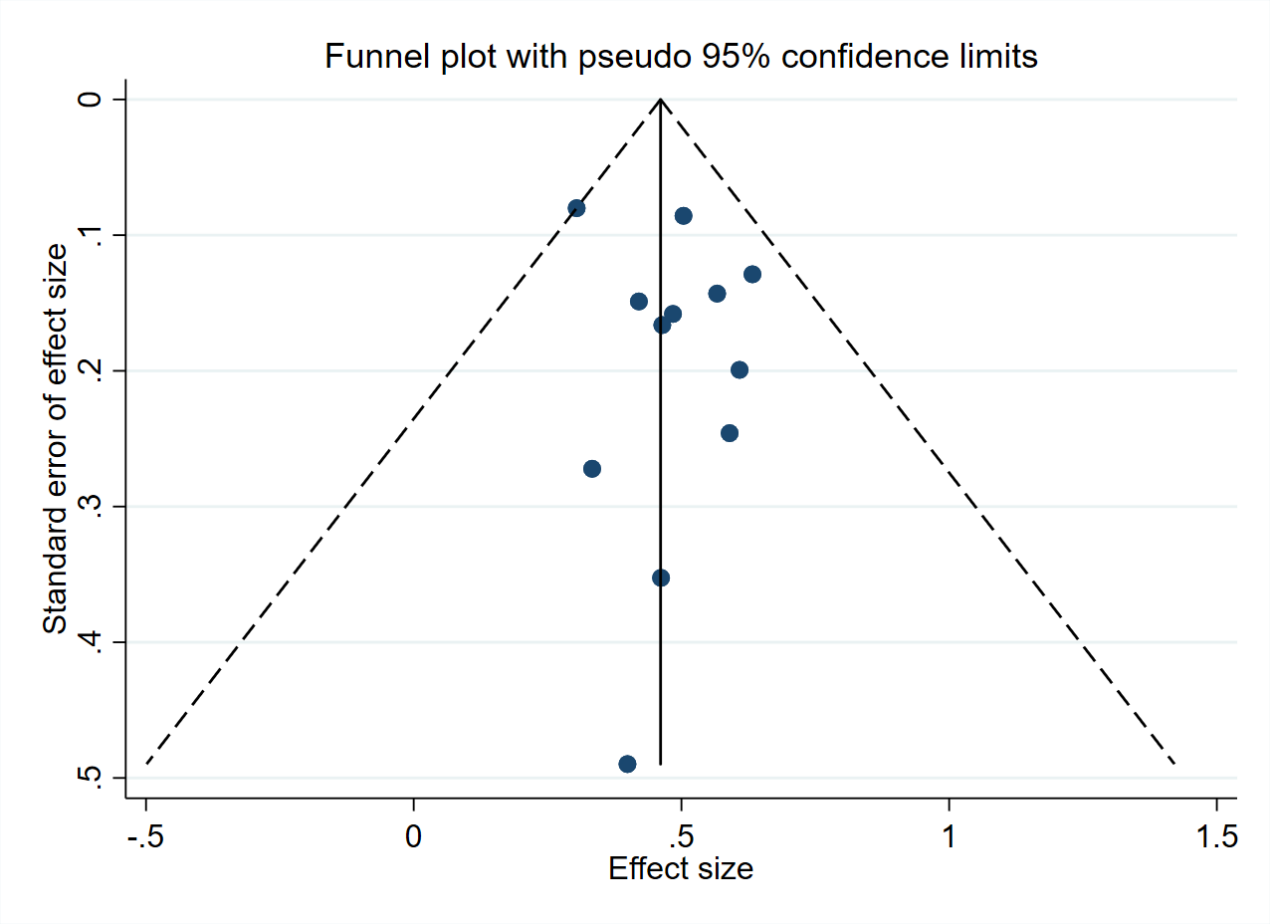
Figure 1S. Sensitivity analysis of the prevalence of oropharyngeal dysphagia in patients after lung transplantation.

Figure 2S. The funnel plot assessed the publication bias for the prevalence of oropharyngeal dysphagia in patients after lung transplantation.


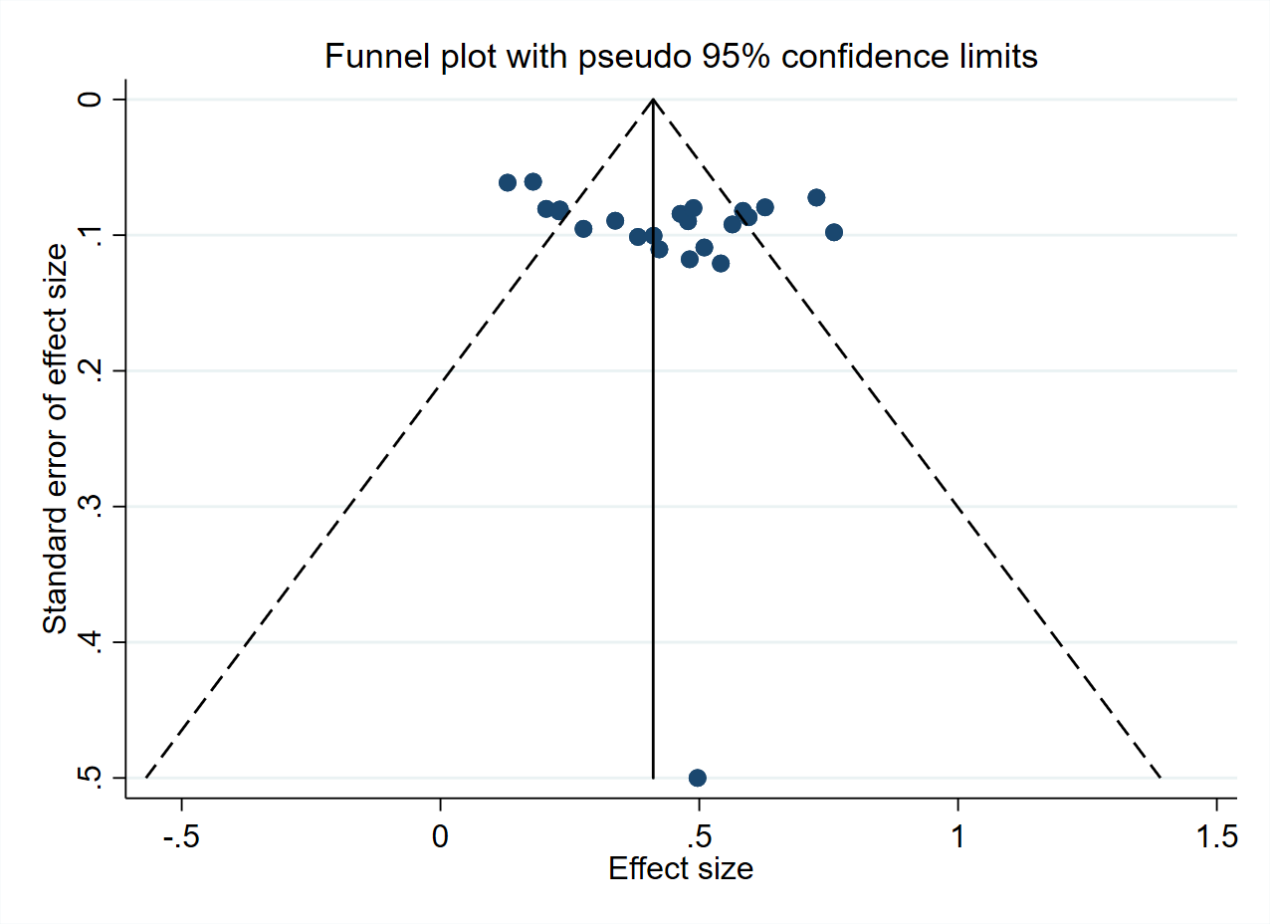
Figure 3S. Sensitivity analysis of the prevalence of gastroesophageal reflux disease in patients after lung transplantation.

Figure 4S. The funnel plot assessed the publication bias for the prevalence of gastroesophageal reflux disease in patients after lung transplantation.

Figure 5S. Sensitivity analysis of the incidence of clinical events between GERD patients and non-GERD patients. COPD, chronic obstructive pulmonary disease; IPF, idiopathic pulmonary fibrosis; CF, cystic fibrosis.
