# Supplementary material for: Oropharyngeal dysphagia and gastroesophageal reflux disease in lung transplant patients: a systematic review and meta-analysis of incidence, risk factors, and clinical outcomes
Source: PeerJ. 2026 Jul 6;14:e21472. doi: 10.7717/peerj.21472 (PMC13348482; doi:10.7717/peerj.21472)
Supplement: Supplemental Information 4 [file peerj-14-21472-s004.docx]

**1. Rationale for Conducting the Meta-Analysis**

Despite advancements in lung transplantation, long-term patient outcomes remain significantly challenged by postoperative complications. Oropharyngeal dysphagia (OPD) and gastroesophageal reflux disease (GERD) have been increasingly recognized as two highly prevalent conditions in this population. Clinically, OPD can lead to aspiration, pneumonia, and malnutrition, while GERD is implicated in the development of bronchiolitis obliterans syndrome (BOS), a major cause of chronic lung allograft dysfunction and graft failure.

Although prior studies have individually reported on OPD or GERD, the existing evidence is characterized by fragmentation, small sample sizes, and considerable heterogeneity in reported prevalence and risk estimates. This lack of a comprehensive, quantitative synthesis has created uncertainty regarding the true magnitude of these problems, their modifiable risk factors, and the strength of their association with critical clinical endpoints. Consequently, there is an urgent need for high-level evidence to inform standardized screening protocols and targeted management strategies. This systematic review and meta-analysis was therefore conducted to definitively quantify the pooled prevalence of OPD and GERD, identify key risk factors for OPD, and evaluate the impact of GERD on pivotal outcomes like BOS, thereby providing a consolidated evidence base to guide clinical practice and future research.

**2. Contribution to Knowledge in Light of Previously Published Reports**

This study provides the first comprehensive and quantitative synthesis specifically focused on the co-occurrence and clinical interplay of OPD and GERD in lung transplant recipients. Its contributions to the field are multifold and address notable gaps in the existing literature:

- **Definitive Prevalence Estimates:** Prior reviews have discussed OPD and GERD qualitatively or reported wide-ranging incidence figures. Our meta-analysis establishes robust, pooled prevalence rates of 46% for OPD and 45% for GERD, offering the most precise quantitative benchmarks to date for understanding the disease burden and allocating clinical resources.
- **Identification of Modifiable Risk Factors for OPD:** While OPD has been documented, evidence on its specific risk factors has been inconsistent. Our analysis moves beyond description to identify strong, modifiable perioperative risk factors. We quantitatively demonstrate that mechanical ventilation increases the odds of OPD by over 5-fold, and reintubation by nearly 3-fold. This evidence directly highlights actionable targets for preventive strategies, such as protocols for early extubation and aggressive dysphagia screening in mechanically ventilated patients.
- **Quantification of GERD’s Impact on Chronic Allograft Dysfunction:** The association between GERD and BOS has been hypothesized but not consistently quantified across studies. Our meta-analysis provides the highest level of evidence to validate this link, showing that GERD is associated with a nearly 3-fold increased risk for developing BOS. This finding powerfully reinforces the imperative for routine GERD evaluation and anti-reflux management post-transplant as a potential strategy to preserve graft function.
- **Clarification of Patient Phenotypes:** The analysis clarifies that cystic fibrosis is a significant patient-level factor associated with a higher incidence of GERD post-transplant. This insight helps in risk-stratifying patients and personalizing surveillance.

In summary, this work consolidates scattered evidence into a clear, evidence-based narrative. It transforms suspected associations into quantified risks, thereby strengthening the rationale for integrating systematic OPD and GERD assessment into the standard of care for lung transplant recipients to ultimately improve survival and quality of life.
